# Validating the RedMIT/GFP-LC3 Mouse Model by Studying Mitophagy in Autosomal Dominant Optic Atrophy Due to the OPA1Q285STOP Mutation

**DOI:** 10.3389/fcell.2018.00103

**Published:** 2018-09-19

**Authors:** Alan Diot, Thomas Agnew, Jeremy Sanderson, Chunyan Liao, Janet Carver, Ricardo Pires das Neves, Rajeev Gupta, Yanping Guo, Caroline Waters, Sharon Seto, Matthew J. Daniels, Eszter Dombi, Tiffany Lodge, Karl Morten, Suzannah A. Williams, Tariq Enver, Francisco J. Iborra, Marcela Votruba, Joanna Poulton

**Affiliations:** ^1^Nuffield Department of Women's and Reproductive Health, University of Oxford, Oxford, United Kingdom; ^2^Sir William Dunn School of Pathology, University of Oxford, Oxford, United Kingdom; ^3^Molecular Biology and Biotechnology, University of Sheffield, Sheffield, United Kingdom; ^4^Centro de Neurociências e Biologia Celular (CNC), Coimbra, Portugal; ^5^UCL Cancer Institute, University College London, London, United Kingdom; ^6^National Heart and Lung Institute, Imperial College London, London, United Kingdom; ^7^School of Optometry and Vision Sciences, Cardiff University, Cardiff, United Kingdom; ^8^Division of Cardiovascular Medicine, Radcliffe Department of Medicine, University of Oxford, Headington, United Kingdom; ^9^Centro Nacional de Biotecnología, CSIC, Madrid, Spain

**Keywords:** mitophagy, mouse model, OPA1, ADOA, mitochondrial fragmentation, high content imaging

## Abstract

**Background:** Autosomal dominant optic atrophy (ADOA) is usually caused by mutations in the essential gene, OPA1. This encodes a ubiquitous protein involved in mitochondrial dynamics, hence tissue specificity is not understood. Dysregulated mitophagy (mitochondria recycling) is implicated in ADOA, being increased in OPA1 patient fibroblasts. Furthermore, autophagy may be increased in retinal ganglion cells (RGCs) of the OPA1^Q285STOP^ mouse model.

**Aims:** We developed a mouse model for studying mitochondrial dynamics in order to investigate mitophagy in ADOA.

**Methods:** We crossed the OPA1^Q285STOP^ mouse with our RedMIT/GFP-LC3 mouse, harboring red fluorescent mitochondria and green fluorescent autophagosomes. Colocalization between mitochondria and autophagosomes, the hallmark of mitophagy, was quantified in fluorescently labeled organelles in primary cell cultures, using two high throughput imaging methods Imagestream (Amnis) and IN Cell Analyzer 1000 (GE Healthcare Life Sciences). We studied colocalization between mitochondria and autophagosomes in fixed sections using confocal microscopy.

**Results:** We validated our imaging methods for RedMIT/GFP-LC3 mouse cells, showing that colocalization of red fluorescent mitochondria and green fluorescent autophagosomes is a useful indicator of mitophagy. We showed that colocalization increases when lysosomal processing is impaired. Further, colocalization of mitochondrial fragments and autophagosomes is increased in cultures from the OPA1^Q285STOP^/RedMIT/GFP-LC3 mice compared to RedMIT/GFP-LC3 control mouse cells that were wild type for OPA1. This was apparent in both mouse embryonic fibroblasts (MEFs) using IN Cell 1000 and in splenocytes using ImageStream imaging flow cytometer (Amnis). We confirmed that this represents increased mitophagic flux using lysosomal inhibitors. We also used microscopy to investigate the level of mitophagy in the retina from the OPA1^Q285STOP^/RedMIT/GFP-LC3 mice and the RedMIT/GFP-LC3 control mice. However, the expression levels of fluorescent proteins and the image signal-to-background ratios precluded the detection of colocalization so we were unable to show any difference in colocalization between these mice.

**Conclusions:** We show that colocalization of fluorescent mitochondria and autophagosomes in cell cultures, but not fixed tissues from the RedMIT/GFP-LC3, can be used to detect mitophagy. We used this model to confirm that mitophagy is increased in a mouse model of ADOA. It will be useful for cell based studies of diseases caused by impaired mitochondrial dynamics.

## Introduction

Mitochondria are important for cells, not just for generating energy, calcium regulation and key biosynthetic processes including synthesis of iron Sulfur centers, but also for apoptosis, signaling, and response to cellular stress (Suomalainen and Battersby, 2018). Mitochondria form a dynamic reticulum in cells, with portions of this network constantly fusing and dividing (Legros et al., 2002). Mitochondrial location and transport are particularly important in neurons, individual mitochondria moving along microtubules in the cytoplasm to synapses and other parts of the cell requiring energy (Li et al., 2004). These dynamics are under the control of molecular players such as Drp1 and Fis1 for fission and mitofusins (Legros et al., 2002) and OPA1 for fusion (Chen and Chan, 2006). Hence mitochondrial diseases are mechanistically diverse and do not necessarily manifest clear evidence of impaired ATP synthesis. For instance, the evidence for respiratory chain dysfunction in mitochondrial optic neuropathies may be very subtle (Yu-Wai-Man et al., 2002). Nevertheless, these are important diseases that impair vision, resulting in lifelong disability.

Exactly how mutations in both mitochondrial DNA (mtDNA), encoding subunits of the respiratory chain and in nuclear genes involved in mitochondrial biogenesis can cause optic neuropathies with rather similar phenotypes is poorly understood. Retinal ganglion cells (RGCs), forming the optic nerve and transmitting visual information to the brain, are the cell type that is affected in both Leber Hereditary Optic Neuropathy (LHON) and Autosomal dominant optic atrophy (ADOA) respectively. Autophagy (a type of cellular quality control) is important for the maintenance of RGCs: even a slight reduction in retinal autophagy levels can alter the capability of RGCs to respond to axonal stress (Boya, 2017) which can be rescued by activating autophagy with rapamycin (Rodríguez-Muela et al., 2012).

ADOA is the commonest inherited optic neuropathy (prevalence 1:25,000) resulting in a bilateral, symmetrical and painless loss of vision, color vision defects, central visual field loss and atrophy of the optic disc. It is a slowly progressive neuropathy, currently irreversible and untreatable. Over 200 mutations in the essential gene OPA1 have been identified, comprising about 60% of patients with ADOA (Yu-Wai-Man et al., 2014). The OPA1 gene encodes a ubiquitous protein involved in mitochondrial dynamics. It plays a crucial role in the regulation of the mitochondrial network and cristae morphology, in oxidative phosphorylation, maintenance of mitochondrial membrane potential (MMP), in apoptosis and in neuronal maturation (Bertholet et al., 2013). Furthermore, upregulation of OPA1 can protect against some types of mitochondrial disease (Civiletto et al., 2015). We showed that mitophagy (a subtype of autophagy for maintaining mitochondrial quality) can be upregulated by OPA1 knock down (Liao et al., 2017). When knock down is profound, mitochondrial DNA (mtDNA) depletion and respiratory chain dysfunction are seen in primary cultures of fibroblasts (Liao et al., 2017) and cortical primary neurons (Bertholet et al., 2013). When OPA1 is over-expressed it can ameliorate defects in the respiratory chain (Civiletto et al., 2015; Varanita et al., 2015). We showed that mitophagy is dysregulated in fibroblasts from patients with either mitochondrial (Dombi et al., 2016) or autosomal inherited (Liao et al., 2017) optic neuropathies. We therefore sought to develop a mouse model to investigate the role of OPA1 in the mitochondrial dynamics of RGCs. We used the B6;C3-Opa1(Q285STOP) mouse which has a heterozygous mutation located in exon 8 immediately before the central dynamin-GTPase domain (Davies et al., 2007). This mutation halves the expression of OPA1 protein in all tissues, including the retina, on Western blot analysis (Davies et al., 2007). Heterozygous mutants show a slow onset of degeneration in the optic nerve, preceded by retinal ganglion cell dendropathy (Williams et al., 2010). Furthermore, the mice demonstrate a reduction in visual function on testing with the optokinetic drum and the circadian running wheel (Davies et al., 2007).

Dysfunctional mitochondria can be recycled by a specific type of autophagy, called mitophagy. The damaged mitochondrial fragment is targeted to a developing autophagosome, called a phagophore, which engulfs the mitochondrion forming a so-called “mitophagosome” (Eid et al., 2016a). This then fuses with a lysosome generating a “mitophagolysosome” (Eid et al., 2016a) that acidifies and degrades its contents, including the mitochondrion. Key stages in this process have been exploited to highlight markers as a readout for mitophagy. For instance, both Finkel (Sun et al., 2015) and Ganley (McWilliams et al., 2016) used genetically encoded fluorescent proteins, targeted to mitochondria, that respond to the drop in pH following fusion with the lysosome (with mKeima and mCherry respectively). These are endpoint and steady state assays, respectively. Because they both depend on lysosomal acidification they are particularly useful for highlighting defects in lysosomal processing, late in the mitophagy process.

We however started our studies before either of these pH sensitive models was available. In studying the effects of OPA1 knock down on mitophagy we postulated that mitochondrial fragmentation might drive mitophagy and hence were specifically interested in engulfment of mitochondria by autophagosomes in the earliest stages of this process. We therefore exploited a tool developed by Mizushima, who visualized autophagosomes by tagging their molecular hallmark, LC3-ll with green fluorescent protein (GFP) (Mizushima et al., 2004). We made mice in which monomeric red fluorescent protein (mRFP) was targeted to mitochondria and crossed these with Mizushima's LC3-GFP mice (Mizushima et al., 2004) to visualize colocalization of mitochondria and autophagosomes. Hence we focussed on an earlier stage of mitophagy than the other two assays (Sun et al., 2015; McWilliams et al., 2016). We developed this as a readout for mitophagy driven by OPA1 knock down, in which we anticipated that excessive mitochondrial fragmentation, apparent in RGCs of this mouse model (Williams et al., 2012), drives mitophagy (Liao et al., 2017).

## Materials and methods

### Media and chemicals

DMEM glucose free (11966-025), DAPI (D1306), and goat anti-rabbit AlexaFluor 488 (A-11008) fluorescent secondary antibodies were purchased from Life Technologies. Primary mouse anti-PDH (sc-377092) antibody was purchased from SantaCruz Biotechnology. DMEM high (4.5g/L) glucose (D6546), Galactose (G5388), and the pharmacological agents E64d (E8640), Pepstatin A (77170), and Chloroquine (C6628) were purchased from Sigma Aldrich. Penicillin and Streptomycin (P4458) were purchased from Sigma Aldrich.

### Genetic modification of mouse embryos

**Figure 1:**
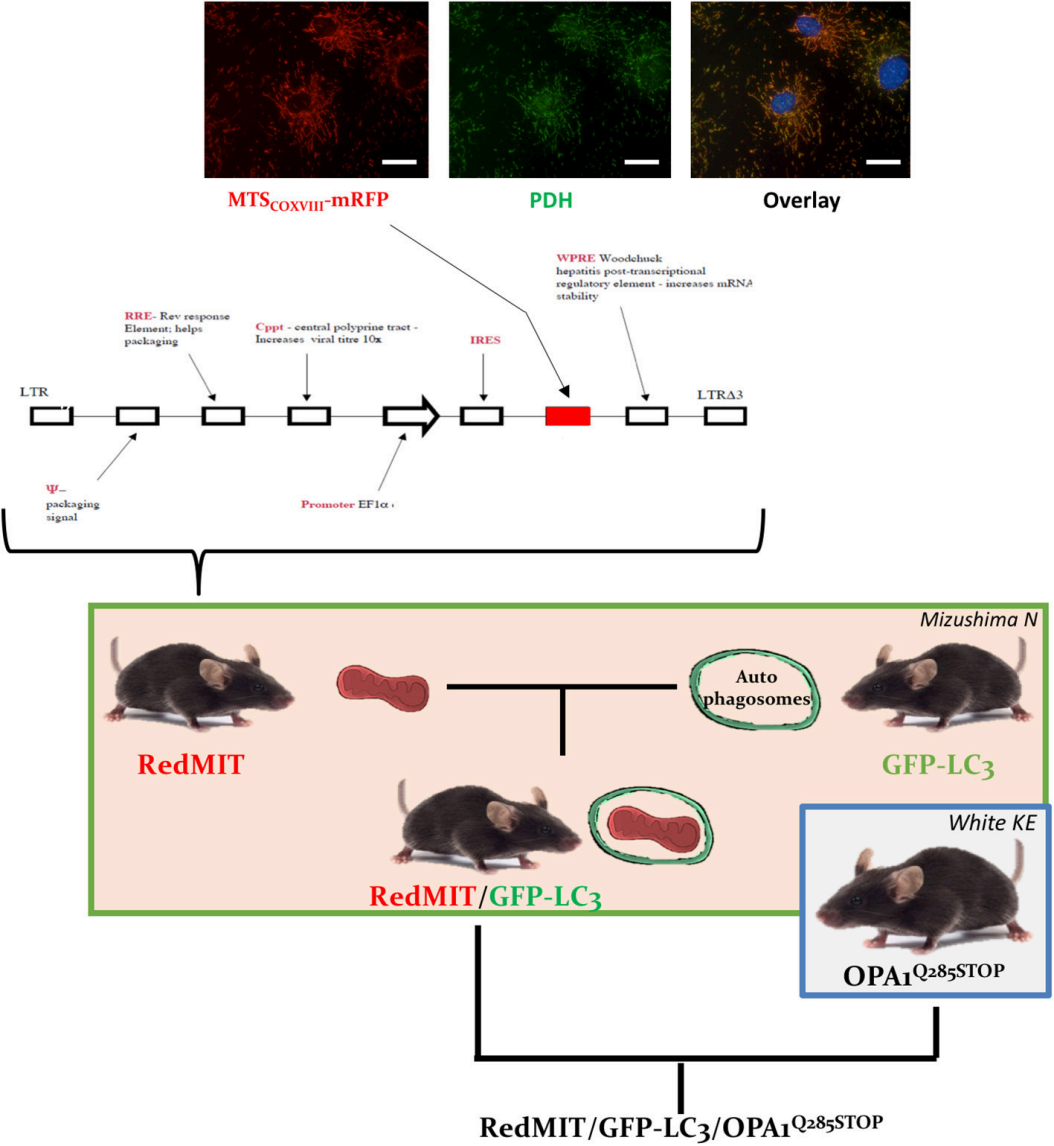
The RedMIT-GFP-LC3-OPA1^Q285STOP^ mouse. The RedMIT mouse has been genetically engineered by random insertion of the illustrated construct. The mRFP fluorescent protein is fused to the mitochondrial targeting sequence of COX VIII and its expression perfectly colocalizes with PDH (panel, scale bar = 20 μm). Expression is under the control of the EF1α promoter. Homozygous females were then crossed to GFP-LC3 males (Mizushima et al., 2004) to obtain our RedMIT-GFP-LC3 mouse model. Once both fluorescent markers were homozygous, RedMIT-GFP-LC3 mice were crossed to the OPA1^Q285STOP^ mice (White et al., 2009).

RedMIT mice, developed by collaborator FI, express mRFP downstream of the COX VIII targeting peptide, driven by the EF1α promoter (ubiquitous expression). Hence mitochondria appear red when viewed by fluorescence microscopy (Figure 1). Mouse ES cells (129 background) were transduced with a VSV pseudotyped pHR'SIN-cPPT-SE lentivirus (Demaison et al., 2002) in which the human EF1a promoter drives expression of a fusion gene containing the mitochondrial localization signal of COX VIII and RFP. A clone with low RFP expression in which viability/function was not impaired by RFP was selected by FACS to produce the mouse by microinjecting ES cells into embryos for founders.

### Mouse embryonic fibroblasts production

A pregnant RedMIT-GFP-LC3 or RedMIT-GFP-LC3-OPA1^Q285STOP^ mouse was sacrificed on embryonic day 13.5 or 14.5 and the uterine horns dissected. The excess fat was trimmed away before opening the uterus carefully to release the conceptuses. The embryonic membranes and placenta were removed from each embryo. The head and internal organs were removed and the remainder minced as finely as possible using a sterile scalpel. It was incubated in trypsin for 5 min at 37°C.

After a vigorous shake to dissociate the tissue, large debris were allowed to settle before the supernatant was transferred to a tube containing DMEM medium + 10% FCS + penicillin/streptomycin (250 μM). The trypsin extraction was repeated three times and the cells split into four 75 cm^2^ flasks. The medium was changed the following day to remove further debris. When the flasks were confluent, each flask was passaged into a 175 cm^2^ flask. Once confluent, these cells were harvested and frozen in 1 ml aliquots at about 10^7^ cells/ml for storage.

### High-throughput microscopy and mitophagy assessment

For IN Cell 1000 analysis, mouse embryonic fibroblasts (MEFs) were seeded at 10^4^ cells per well in a Nunc F 96-well plate (Thermo Scientific) and treated in the indicated conditions for 24 h. Cells were fixed with 4% (w/v) paraformaldehyde (PFA) for 15 min before DAPI staining (dilution 1/5,000 for 5 min). The cells were imaged using the IN Cell 1000 analyzer (500 cells acquired per well) and raw images processed and parameters obtained using a previously published (Diot et al., 2015) customized protocol in the IN Cell Developer toolbox (GE Healthcare Life Sciences).

We used Imagestream, which we previously validated for detecting mitophagy (Liao et al., 2017), for analysis of splenocytes. Spleens were removed immediately after death and placed in individual sterile dishes with 1 mL of cell culture media. The spleen cells were separated out by mechanical disruption and divided equally between two 25 cm^3^ flasks in 5 mL of DMEM. Chloroquine was added to a concentration of 20 μM to one of the flasks and both flasks incubated overnight at 37°C. Floating and trypsinized cells were filtered through a 30 μm filter and fixed in 4% PFA for 15 min at room temperature. After permeabilization in 0.4% Triton for 3 min, cells were washed in PBS and resuspended in 50–100 μL of FACS buffer for analysis with Imagestream (1,000–5,000 cells in each condition from three mice in each condition).

To identify colocalization of autophagosomes and mitochondria as an indicator of mitophagy we used Amnis IDEAS software, counting the numbers of LC3 positive puncta that colocalized with mitochondria, using a “threshold” mask for detecting mitochondrial location. Duplicate analyses using threshold masks of either 30 or 70% of the range of intensity values as defined by the starting mask was used to exclude pixels.

### Oxygen consumption measurement

MEFs were plated at a density of 50,000 per cells per well in black 96 well plates with clear bottoms (Falcon Corning). Cells were left for 7 h to attach and then media switched to media with glucose (25 mM) or galactose (10 mM) and incubated in a standard 37°C −5% CO_2_ incubator. A parallel plate was set up for Hoechst quantification to allow normalization for cell number. After 16 h media were replaced with fresh media containing the MitoXpress xtra oxygen probe (Luxcel Biosciences) and overlayed with mineral oil. The oxygen consumption assay was carried on in a BMG OMEGA plate reader equilibrated at 37°C and monitored for at least 4 h. Initial oxygen consumption rates (fluorescence life time) were calculated in the linear phase of the assay and standardized to cell number measured using Hoechst on the parallel plate.

### Mouse husbandry and ethics statement

All animals were housed and managed in accordance with the United Kingdom's Home Office protocols, covered by the Animals (Scientific Procedures) Act 1986. The protocol was approved by the Oxford University Committee on Animal Care and Ethical Review, University of Oxford Medical Sciences division (Project licenses 3002208 and 3003213).

Mice were housed in conventional wire-top polycarbonate cages, with a 12:12 light:dark cycle at temperatures between 19 and 23°C and relative humidity 55 ± 10%. Food and water were offered ad libitum. The facility is free of *MHV, EDIM, MVM, MPV, PVM, Sendai, TMEV, ectomelia, LCMV, Mad 1, and 2, MCMV, reovirus 3, Citrobacter rodentium, Clostridium piliforme, Corynebacterium kutscheri, Mycoplasma, Pasteurellaceae, Salmonella, beta-hemolytic streptococci, Streptococcus pneumoniae, Streptobacillus moniliformis, endoparasites*, and *ectoparasites. Helicobacter* and *MNV* are present in this facility.

### Confocal microscopy

MEFs were plated onto 0 thickness coverslips and treated as described in the main text. Four percent paraformaldehyde was used for fixation (10 min, room temperature). Cells were permeabilized and washed in 0.1% Triton-Tris buffered saline three times before mounting on slides using Vectashield (Vector Labs). Images were acquired on an upright Leica SP5 confocal microscope equipped with the appropriate filters and sequential 488, and 568 nm laser illumination.

For mouse eyes, four samples (2x RedMIT-GFP-LC3 and 2x RedMIT-GFP-LC3-OPA1^Q285STOP^) were harvested from perfused-fixed mice and cryostat eye sections cut at 10 μm, lightly counter-stained with DAPI (1:30,000 dilution; 5 min) and sealed using aqueous glycerol-based mountant with a No. 1.5 coverslip. They were examined using a Zeiss LSM 700 inverted confocal microscope with a plan-Apo 63x NA 1.4 oil-immersion objective. The optical section thickness was set at 1.0 micrometer, and as far as was practicable the optic nerve head was examined. The maximum Pearson product moment correlation coefficient values were recorded across all four slides, using the colocalization software in the Zeiss Zen Black, Zen 2.3 SP1 version 14.0.0.0.0 with the scatter-plot threshold set to three times the standard deviation of the mean value of the background pixels, as investigated by Barlow et al. (2010).

### Live cell imaging

MEFs, cultured and treated as described above, were plated into a 35 mm MatTek dish, and supplemented with 10 mM Hepes to buffer pH during live cell imaging. A custom Olympus IX81 inverted microscope equipped with temperature control (Solent scientific), LED illumination (Cairn Research), a Semrock quad-band filter set (bandpass filter (DS/FF01-387/485/559/649-25), dichroic quad-edge beam splitter (DS/FF410/504/582/669-Di01-25x36), and quad bandpass emission filter (DS/FF01-440/521/607/700-25) and simultaneous dual image acquisition with two C-1900 EMCCD's (C1900, Hamamatsu, Japan) mounted after a beam splitter (Dual View C2, Photometrics) controlled thorough CellR (Olympus, Japan) with a x60 oil immersion lens (Olympus, NA 1.42). Simultaneous red and green images were acquired as a z-stack every 30 s to enable subsequent Weiner filter deconvolution (CellR). A single z-plane is selected for the overlay time series shown with post-processing (time stamping and compression) for publication using Videomach (gromeda.com).

## Results

### Engineering the RedMIT-GFP-LC3 mouse

In order to visualize mitochondrial fate, we first generated a mouse expressing mRFP fused to the COX VIII mitochondrial targeting sequence (Figure 1), engineered by random insertion into embryonic stem cells. Mice that were homozygous for the insert, located in the *pkn1* gene (Figure S1) appear to be normal with no noticeable effects observed on lifespan or litter size.

**Figure 2:**
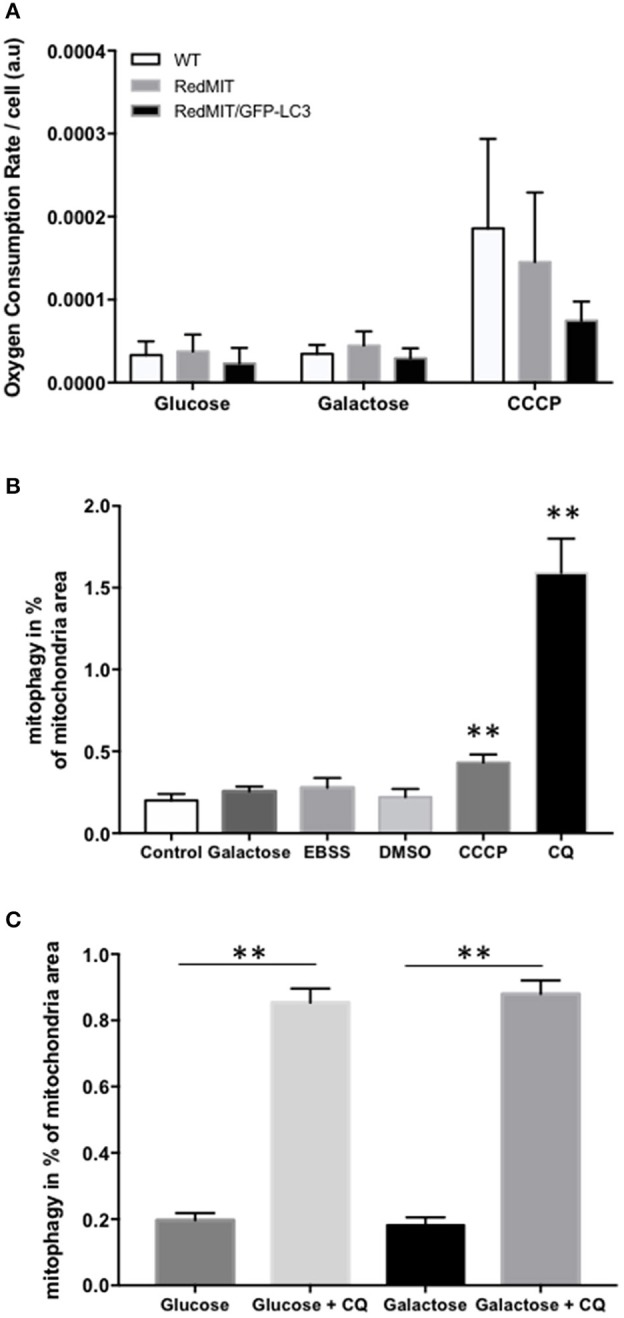
Validation of the RedMIT-GFP-LC3 Mouse model for detecting mitophagy (see Diot et al., 2015 for detailed validation of high throughput imaging to quantify mitophagy). **(A)** MEFs from WT, RedMIT and RedMIT-GFP-LC3 mice were used to clarify whether expressing mRFP in mitochondria impaired their function. No significant differences in oxygen consumption were observed either in glycolytic (Glucose), oxidative (Galactose), or uncoupled (CCCP) conditions [mean + SD, *n* = 2, *t*-test *p* = 0.625–0.684–0.289 for wt vs. RedMIT-GFP-LC3/0.832–0.559–0.714 for wt vs. RedMIT/0.532–0.409–0.369 for RedMIT vs. RedMIT-GFP-LC3 (glucose—galactose—CCCP, respectively)]. **(B)** Using our IN Cell 1000 high-throughput microscopy assay (Diot et al., 2015) we validated detection of mitophagy in RedMIT-GFP-LC3 MEFs grown for 2 h in glucose media (control), glucose-free galactose-based media (galactose), starvation media (EBSS), media containing vehicle (DMSO), glucose media supplemented with 10 μM CCCP (CCCP), and glucose media supplemented with 25 μM chloroquine (CQ). After fixation, an increase colocalization between mitochondria and LC3 was observed when using an inhibitor (chloroquine, CQ) of mitophagy, and when using CCCP compared to DMSO control (mean + SEM, at least 500 cells counted per condition, **p < 0.01, *t*-test). **(C)** Using IN Cell 1000, chloroquine significantly increased colocalization of mitochondrial fragments with autophagosomes expressed as a proportion of mitochondrial area in both regular (glucose) and glucose-free galactose based media (**p < 0.01, *t*-test, effect of medium NS, error bars are SE at least 500 cells counted).

We confirmed the mitochondrial localization of mRFP in MEFs derived from these mice (Figure 1) and observed an O_2_ consumption similar to that of wild type MEFs, suggesting that genetic modification had not significantly affected mitochondrial function (Figure 2A).

To visualize mitochondria specific autophagy, i.e., mitophagy, we crossed this mouse model with the previously described mouse expressing LC3, the hallmark of autophagosomes, tagged with GFP protein (Mizushima et al., 2004). This double fluorescent labeling had no noticeable effect on mice, either reproduction (Table S1), lifespan or litter size.

Then we generated MEFs and imaged them using the high throughput imaging system employed previously (Diot et al., 2015) to explore mitochondrial dynamics, autophagy and mitophagy in this mouse model (Figures 2B,C). Like oxygen consumption (Figure 2A), cell growth (not shown) was not affected by the expression of the two fluorescent markers. We routinely CCCP to uncouple mitochondria as a positive control for oxygen consumption. Given the non-significant trend to lower uncoupled respiration in the RedMit/LC3-GFP MEFs, we have not excluded a subtle defect.

MEFs were treated with CCCP (carbonyl cyanide m-chlorophenyl hydrazone) to induce mitophagy or with chloroquine to block the final step of the autophagy pathway and analyzed using our IN Cell system (Figure 2B). We showed that an induction of mitophagy, or a block in the late stages of mitophagy both result in an increase in the mitophagy signal detected (colocalization of mitochondrial fragments with autophagosomes, Figure 2, *p* < 0.05), consistent with previous results (Diot et al., 2015).

As with other cell types, MEFs responded to growth in glucose-free galactose media and starvation media by an induction of mitophagy and mitochondrial fragmentation (Malik et al., 2017). Because the basal level of mitophagy in glucose-free, galactose-based media, is frequently higher than in media containing glucose (but NS in Figure 2B) we run our assay in both regular and galactose-based media. Using chloroquine to inhibit lysosomal acidification (25 μM for 16 h) thus blocking the late steps of the auto/mitophagy pathway, we induced a similar accumulation of mitochondria colocalising with autophagosomes in galactose and glucose- containing media (Figure 2C). Data were normalized by log transformation prior to ANOVA. Chloroquine significantly increased colocalization of mitochondrial fragments with autophagosomes expressed as a proportion of mitochondrial area (*p* < 0.01, effect of galactose NS).

**Figure 3:**
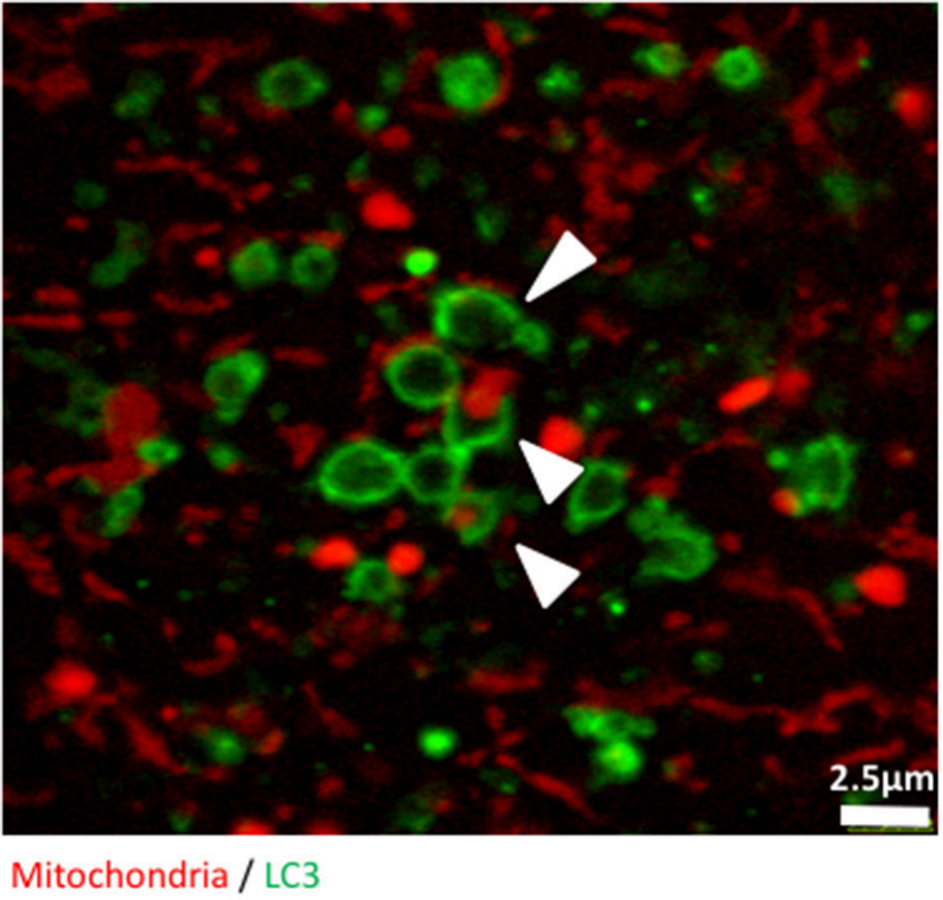
The early stages of mitophagy can readily be demonstrated in RedMIT-GFP-LC3 MEFs. RedMIT-GFP-LC3 MEFs were cultured in glucose-free galactose media (galactose). After fixation, the cells were imaged using a Leica SP5 confocal microscope with a 63X lens and further digital zoom when needed. Autophagosomes forming around a mitochondrion are observed under these “energetic stress” growth conditions. The white arrowheads show different phases of autophagosome formation (green) around mitochondrial cargo (red). Similar observations in whole cells images have been made in Liao et al. (2017).

Finally, we used confocal microscopy to confirm and illustrate our observations with better quality pictures than the ones produced with the high throughput imaging system. Figure 3 illustrates autophagosomes engulfing mitochondria in cells grown in glucose-free galactose media. These cells can be monitored in real time using time lapse imaging, as we did not observe any significant bleaching of the fluorescent signals when the cells were imaged every 30 s for 8 h, enabling tracking of mitophagy in living cells (Video S1).

Together these results confirm that MEFs from the RedMIT-GFP-LC3 mouse are a useful model for the *in vitro* assessment of mitochondrial dynamics.

### The RedMIT-GFP-LC3-OPA1^Q285STOP^ mouse

**Figure 4:**
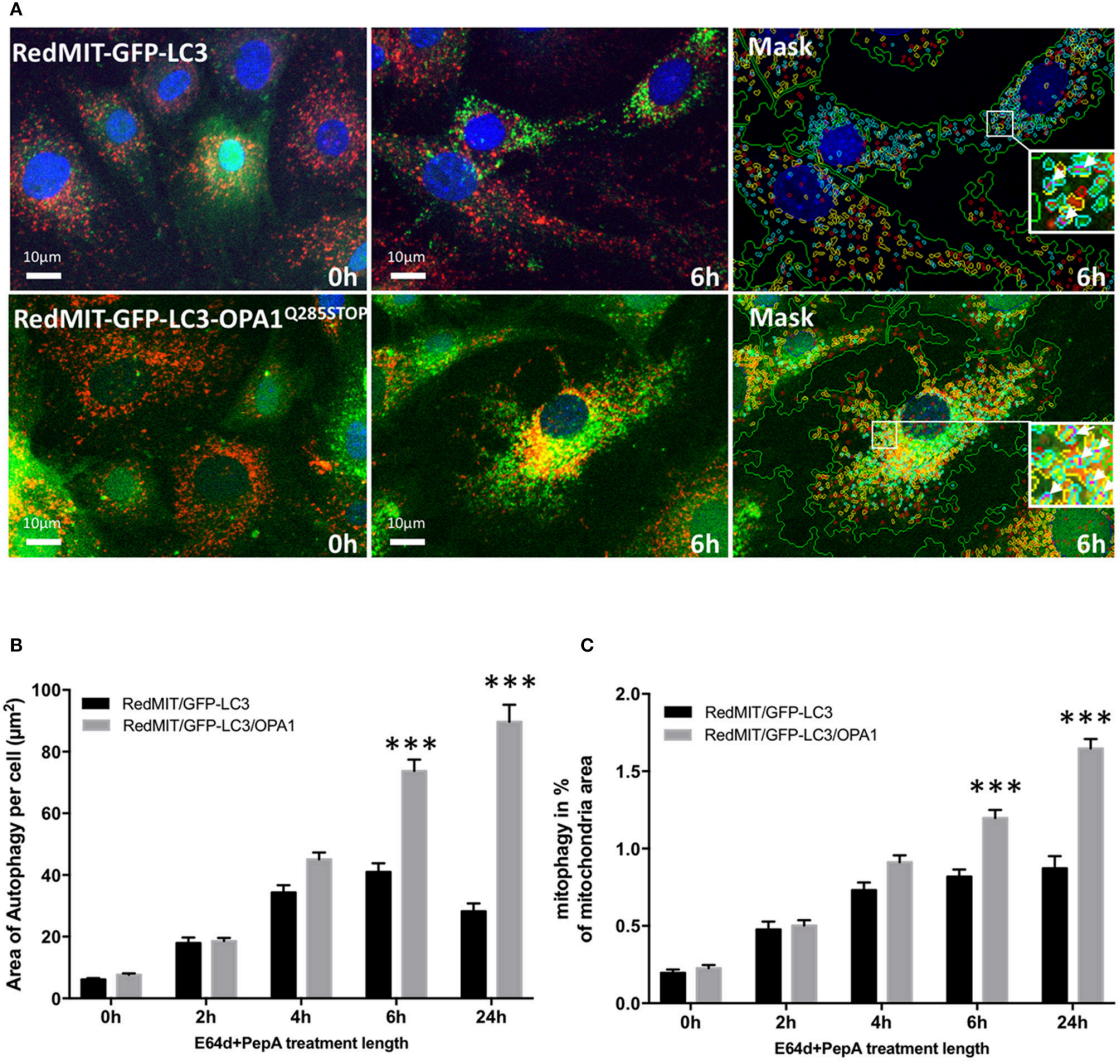
The OPA1^Q285STOP^ mutation induces mitophagy in MEFs. **(A)** Representative images of the IN Cell 1000 high content imaging acquisition system. The cells from both RedMIT-GFP-LC3 and RedMIT-GFP-LC3-OPA1^Q285STOP^ were grown in glucose media (0 h) supplemented with E64d/Pepstatin A for 6 h before fixation. The images are analyzed using a homemade protocol developed using the IN Cell developer toolbox (Diot et al., 2015) resulting in a “mask” picture (cyan: autophagosomes; red: “short” mitochondria; yellow: “long” mitochondria; purple: “colocalization” between autophagosome and mitochondria signals). The white arrows in the insets indicate the colocalization events between mitochondrial and autophagosome signal. **(B)** Lysosomal inhibitors E64d/Pepstatin A were added to cells growing in glucose media to block the processing of autophagolysosomes. As shown on the graph a greater accumulation of autophagosomes is observed in MEFs from the RedMITGFP-LC3-OPA1^Q285TOP^ mouse compared to the RedMIT-GFP-LC3-OPA1^+/+^ mouse, indicating a greater flux of autophagy when the OPA1 mutation is present. (at least 500 cells counted, regression *p* < 0.05). **(C)** In similar conditions, a greater flux of mitophagy (mitochondrial fragments colocalizing with autophagosomes) is observed with the OPA1 mutation: the rate of accumulation of mitophagosomes is 0.73 ± 0.21 (SEM) for wild type and 1.96 ± 0.22 for OPA1 (at least 500 cells counted, regression *p* < 0.02). ***p < 0.001 at specified time points.

We previously showed that mitophagy is increased in fibroblasts from patients with bi-allelic OPA1 mutations (compound heterozygotes Liao et al., 2017). In order to understand the mechanisms underlying this mitophagy dysregulation we crossed our RedMIT-GFP-LC3 mouse with a model of the DOA disease, the OPA1^Q285STOP^ mouse. It is already known that autophagosomes are increased in RGCs in the OPA1^Q285STOP^ mouse (White et al., 2009) and electron microscopy suggests increased mitophagy (Sarzi et al., 2012). But to our knowledge, it is not yet clear whether mitophagic flux is increased. We previously observed increased mitophagy in fibroblasts from ADOA patients in whom we had demonstrated OPA1 deficiency, and therefore wished to explore whether mitophagy was increased in the OPA1^Q285STOP^ mouse model, potentially explaining the increased autophagosomes in RGCs. We generated RedMIT-GFP-LC3-OPA1^Q285STOP^ MEFs, to investigate the effects of OPA1 knock down on autophagy and mitophagy (Figure 4). We confirmed that the abundance of OPA1 in the resulting MEFs (Figure S2) was lower than in OPA1 wild type MEFs, as expected (Davies et al., 2007). Increased counts of colocalized mitochondria with autophagosomes in the OPA1 ± MEFs (Figure 4A) suggested increased mitophagy. Because this increased colocalization could be due to either activated mitophagy or slowed turnover we assessed mitophagic flux by adding lysosomal inhibitors. We thus blocked the late steps of the autophagy pathway using E64d and pepstatin A over a time-course of 24 h (Figure 4B).

The accumulation of autophagosomes (expressed as the summed area of LC3-ll positive punctae per cell) is significantly greater in the OPA1 mutant compared to the OPA1 wt (*n* = 3) as shown in Figures 4A,B. This increase was significant (*p* < 0.01, Data were normalized by log transformation prior to ANOVA). As the area of autophagy detected is similar in both the OPA1 wild type and mutant at baseline, but is greater in the OPA1 mutant following inhibitors, this indicates that in steady state conditions, the flux of autophagy is increased in the OPA1 mutant cells compared to the wild type. Similarly, the lysosomal inhibitors significantly increased colocalization of mitochondrial fragments with autophagosomes expressed as a proportion of mitochondrial area (Figure 4C) in the OPA1 mutant cells (*p* < 0.01, effect of galactose NS). This result is consistent with our previous results in fibroblasts from patients with bi-allelic OPA1 mutations of OPA1 (Liao et al., 2017).

Together these results show that the OPA1^Q285STOP^ mutation dysregulates both autophagy and mitophagy. Hence this mouse model is useful for studying the underlying mechanisms of the dysregulated mitophagy induced by OPA1 dysfunction in isolated cells.

### Investigation of fixed mouse splenocytes

**Figure 5:**
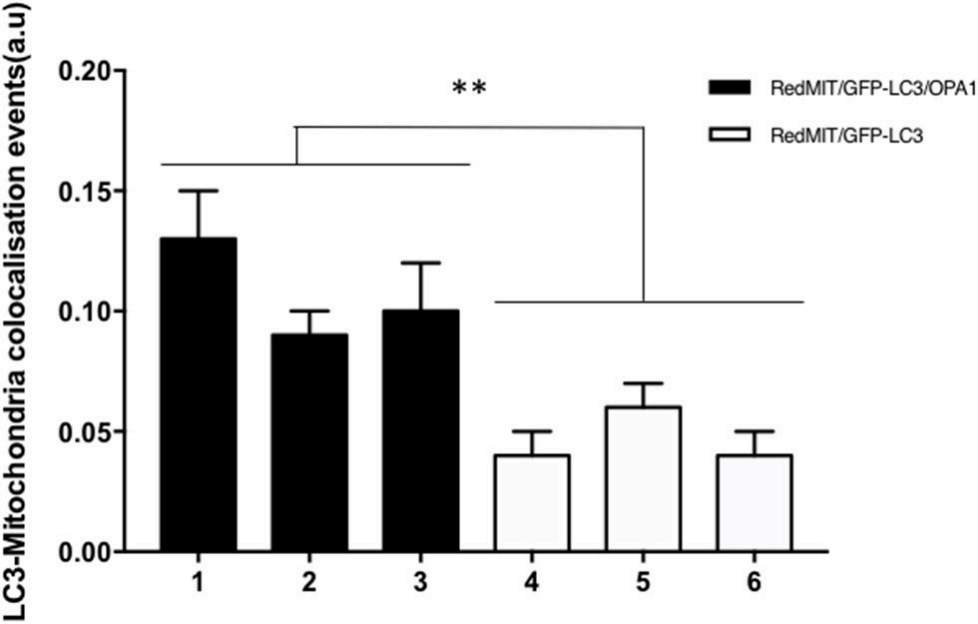
Baseline mitophagy is increased by the OPA1^Q285STOP^ mutation in mouse splenocytes. Fixed splenocytes (1,000–5,000 per mouse) from three RedMITGFP-LC3-OPA1^Q285TOP^ mice (designated 1–3) and three RedMIT-GFP-LC3 mice (designated 4–6) mice were prepared and analyzed using the Imagestream (Amnis) system, error bars are SE of technical replicates. In line with the results observed in MEFs, colocalization between mitochondria and LC3 is increased in the OPA1 mutant mice. (*t*-test, **p < 0.01).

In order to assess these effects in whole organisms we investigated mouse splenocytes, since large numbers of cells can readily be harvested. This time we used Imagestream, another high content imaging system, in which FACS is coupled to a fluorescence microscope, to investigate colocalization of mitochondria and autophagosomes. We had previously validated this method for detecting mitophagy in human fibroblasts (Liao et al., 2017). Briefly, the sorted and individualized splenocytes in the flow cytometer are imaged with an integrated fluorescence microscope. The pictures are then analyzed using masks and the colocalization between mitochondria and autophagosomes assessed. Again, we demonstrated a significantly higher level of colocalization in OPA1 mutants compared to OPA1 wild type (*p* < 0.01, Figure 5). This result confirms the induction of mitophagy by the OPA1^Q285STOP^ mutation in the mouse. We therefore investigated the organ mainly affected by dysregulated mitophagy, i.e., the retina.

### Mouse retina investigation

**Figure 6:**
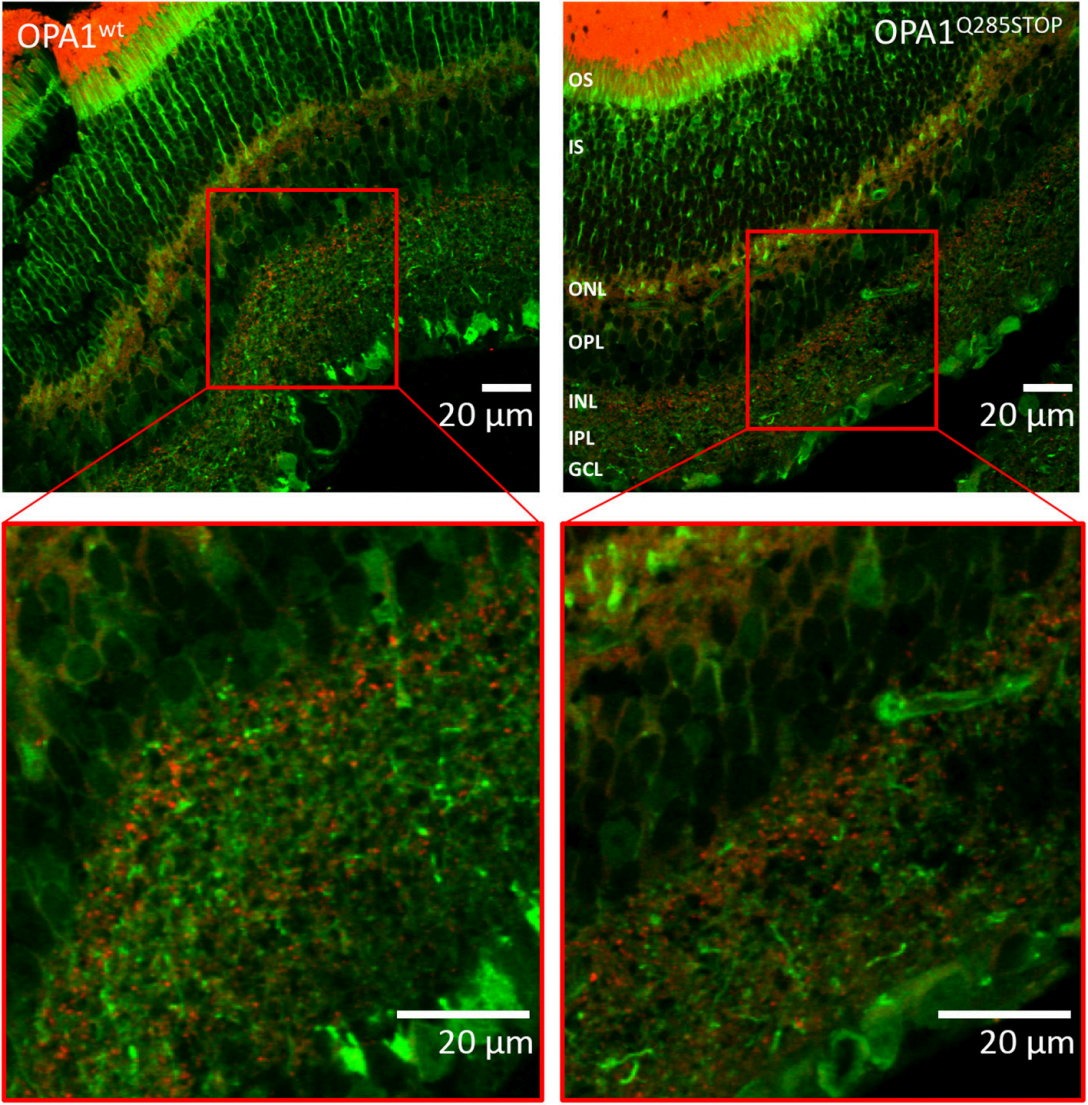
Confocal images of transgenic OPA1wt and OPA1 Q285STOP mouse retina sections expressing LC3-GFP and RedMIT Representative images of the retina from the RedMIT-GFP-LC3-OPA1^+/+^ **(Left)** and the RedMIT-GFP-LC3-OPA1^Q285STOP^ **(Right)** mice sections show autophagosomes with GFP-tagged LC3 and RedMIT tagged mitochondria. Sections were cut at 10 μm and visualized using a Zeiss LSM 700 inverted confocal microscope with a plan-Apo 63x NA 1.4 oil-immersion objective. Red boxes indicate the area magnified in each inset. RedMIT were observed from the ONL toward the IPL in both OPA1^wt^ *and* OPA1 ^Q285STOP^. No difference in GFP-LC3 or mitochondrial mRFP is seen between OPA wild type and mutant (zoomed insets, bottom panels). In particular, no colocalization was observed between GFP-LC3 and RedMIT in both OPA1^wt^ *and* OPA1^Q285STOP^ (Pearson correlation coefficient of +0.22 and −0.16; *n* = 4). Scale bars: 20 μm OS, outer segments; IS, inner segments; ONL, outer nucleus layer; OPL, outer plexiform layer; INL, inner nucleus layer; IPL, inner plexiform layer; GCL, ganglion cell layer.

As DOA affects the RGCs we investigated autophagy and mitophagy in the retina. For this, four samples from the RedMIT-GFP-LC3-OPA1^Q285STOP^ mice and the RedMIT-GFP-LC3 control mice were harvested after fixation by perfusion. Cryostat eye sections were cut at 10 μm and examined using a Zeiss LSM 700 inverted confocal microscope with a plan-Apo 63x NA 1.4 oil-immersion objective. The GFP, which was expected to be targeted to autophagosomes, was homogenously and non-specifically expressed throughout the retinal tissue, as was the mRFP fluorescent mitochondrial signal (Figure 6). Furthermore, the expression levels of fluorescent proteins were weak. The results from each slide were very similar; it was not possible to conclude that colocalization between the GFP and mRFP occurred, as the image signal-to-background ratios precluded the detection of colocalization with a Pearson correlation coefficient values being either very weakly negative, or very weakly positive. Indeed, the maximum Pearson product moment correlation coefficient values recorded across all four slides were +0.22 and −0.16. We did not detect consistent differences in other tissues examined (Figure S3). In summary, the high background and the weak red transgene expression precluded any significant colocalization analysis.

## Discussion

### Mouse model demonstrates increased mitophagy in primary cell cultures

We have developed a mouse with genetically encoded fluorescent proteins mRFP and GFP directed to mitochondria and autophagosomes respectively (Figure 1). We have shown that colocalization of these tags can be used as a readout for mitophagy in live cells (Figure 2), using two different high throughput imaging systems (Figures 4, 5). In live cells from the well characterized mouse model of ADOA (OPA1 ^Q285STOP^) (Davies et al., 2007) which develops an adult-onset dendropathy and impaired vision, we have shown that baseline mitophagy is increased in splenocytes (Figure 5) and mitophagic flux is increased in MEFs (Figure 4). Colocalization of mitochondria with autophagosomes was investigated in retina using confocal microscopy (10 μm cryostat sections).

These data validate both our model and our high throughput imaging method for quantifying mitophagy. They are consistent with our suggestion that OPA1 knock down causes an excessive mitochondrial fragmentation, and that this activates mitophagy (Liao et al., 2017).

### This model visualizes an earlier stage in mitophagy than do other mouse models

There are considerable technical difficulties with visualizing mitophagy, because it is transient and occurs at low frequency. We set up the high throughput imaging system for quantifying mitophagy in cells (Diot et al., 2015) that we have used in this study. In order to study the mechanisms underlying pathogenesis further, with a view to testing therapeutic strategies *in vivo*, we decided to develop a mouse model allowing us *in vivo* imaging of mitophagy. All three models employed to date use genetic manipulation to express fluorophores (Sun et al., 2015; McWilliams et al., 2016). While two of these can be used both in live and fixed cells, the mt-Keima is not compatible with fixation. The RedMIT-GFP-LC3 mouse model visualizes mitochondrial fragments engulfed by autophagosomes, at an earlier time point in mitophagy than the other mouse models that use mt-Keima and mCherry, which demonstrate fusion with lysosomes. Our model thus complements the previously published models (Sun et al., 2015; McWilliams et al., 2016), which are steady state and endpoint assays, respectively. Both the mt-Keima and the mCherry constructs are functionally inert, but our GFP tag is linked to expression of LC3, an important part of the mitophagy process. Furthermore, our study is limited by our use of the EF1α promoter which drives expression of mRFP. While this promoter is suitable for studies of developing embryos, being ubiquitous in most cell types (Chambers et al., 1998), it is less suitable for studies of post-mitotic tissues such as muscle. Hence the mitochondria of post-mitotic tissues were poorly visualized using mRFP alone, after post-natal day 40.

While we are able to demonstrate increased mitophagic flux in primary cultures, it is not easy to demonstrate this in whole animals using any of the existing models (Sun et al., 2015; McWilliams et al., 2016).

### Microscopic examination of tissue sections added no support to data from primary cultures

Despite excellent technical input, we were unable to visualize the colocalization of mitochondria and autophagosomes, that was apparent in MEFs and splenocytes, in cryostat sections of retina or other tissues. We consider three possible explanations for this. Firstly, our model is not sufficiently sensitive. On fusion with lysosomes, GFP is bleached by a drop in pH. Colocalization of the GFP and mRFP signals is therefore short lived. This appears less problematic in cultured cells, where levels of mitophagic flux can be increased by activators, than it is in fixed tissue sections, where colocalization events are less frequent and the signal-to-background ratio sub-optimal. Secondly there are many different subtypes of mitophagy (Lemasters, 2014) and we do not yet know whether the read-out of our high throughput imaging method, that increases with OPA1 knockdown (Liao et al., 2017) is BNIP3 dependent. Thirdly, it is possible that mitophagy is not actually increased in RGCs in OPA1 knock down. Indeed, Belenguer showed that BNIP3 dependent mitophagy is actually decreased in a neuronal model of OPA1 knock-down (Moulis et al., 2017). Given that we previously showed that mitophagy declines with maturity in fibroblasts (Diot et al., 2015), we suggest that their neuronal cultures may not reflect *in vivo* mature RGCs.

### The importance of activated mitophagy in ADOA

Up until now, much of the evidence for increased mitophagy in ADOA has been indirect. Electron microscopy of RGCs is consistent with increased autophagy (White et al., 2009). OPA1 knock down in cultured primary cortical neurons impairs maturation, resulting in reduced mtDNA, and abundance of cytochrome oxidase (Bertholet et al., 2013), consistent with, but not attributed to, activated mitophagy. Using the same system other authors have suggested that BNIP3-dependent mitophagy may be decreased rather than increased in OPA1 knock down (Moulis et al., 2017), but these authors did not measure mitophagic flux. We postulate that the increase in mitophagy that we have demonstrated may be different from the BNIP3-dependent type studied by Belenguer (Moulis et al., 2017) as we have never detected accumulation of Parkin. The latter appears to require profound depolarization to a level that does not occur *in vivo*. Furthermore, it has become increasingly clear that there are more than one, and potentially several different types of mitophagy. Some of these are dependent on Eid et al. (2016b), and others are independent of, PINK1/Parkin (Lemasters, 2014). Indeed, recent data show that PINK1/Parkin knockdown do not diminish basal mitophagy, even in dopaminergic cells, in ether mice (McWilliams et al., 2018) or flies (Lee et al., 2018). Hence their role may be confined to specific stresses (Eid et al., 2016b) and/or differentiation (Sarraf and Youle, 2018).

The severe phenotypes caused by mutations in genes regulating mitochondrial dynamics highlight the importance of mitochondrial fusion and fission in maintaining cellular, particularly neuronal, health (Schwarz, 2013; O'Mealey et al., 2017). Many disorders of mitochondrial dynamics involve neurodegeneration, including central (Ryan et al., 2015; Dombi et al., 2016; Haack et al., 2016) and peripheral nervous system (Züchner et al., 2006; Liao et al., 2017) as well as severe malformation syndromes (Cullup et al., 2013). While mechanisms of cellular aging are clearly important (Diot et al., 2016; Lang et al., 2017), the precise cause of the neurodegeneration is rarely clear.

While increased mitophagy may exert effects on local energy supply within the cell, it is increasingly apparent that significant mitochondrial stresses can be signaled to other parts of the cell. For instance, the UPRmt is a stress response pathway acting as a “checkpoint” for mitochondrial fitness that signals the nucleus (Callegari and Dennerlein, 2018). Acute and chronic mitochondrial respiratory chain deficiency differentially regulate lysosomal biogenesis (Raimundo et al., 2016). Signaling of the acute response requires both TFEB and AMPK. Given that OXPHOS deficiency also results in AMPK-dependent mitochondrial fragmentation (Toyama et al., 2016), mitochondrial dynamics could contribute to the lysosomal response. In the case of OPA1 (Sarzi et al., 2018), mitochondrial fragmentation is apparent before loss of dendrites in RGCs while MMP is maintained (Williams et al., 2010). Hence delivery of mitochondria to the regions of the cell requiring energy may be as important as mitochondrial quality. OPA1 levels affect mitochondrial membrane cristae structure (Alavi et al., 2007) and potentially impact on apoptosis, though this in not apparent in fibroblasts (Liao et al., 2017). Others have suggested that OPA1 might be important for mtDNA maintenance (Elachouri et al., 2011) but it does not appear to be a component of the nucleoid. We documented mtDNA depletion in OPA1 knock down and suggest that this is caused by excessive mitochondrial fragmentation increasing mitophagy beyond the level that it is able to maintain mitochondrial quality. This may recapitulate the excessive mitophagy seen in fibroblasts treated with phenanthroline. This treatment depleted mtDNA copy number to 15% of baseline along with a halving in mitochondrial mass without a significant benefit to the quality of mtDNA (Diot et al., 2015). Activated mitophagy may thus become excessive in its demands on mitochondrial biogenesis, by potentially impairing the ability of the RGCs to generate sufficient mitochondria for dendritic growth and/or response to stress.

## Conclusion

In conclusion, we developed the RedMIT-GFP-LC3 mouse model in which colocalization of fluorescent mitochondria and autophagosomes can be used as a readout to detect mitophagy. We used this model to confirm that mitophagy is increased in cell cultures of a mouse model of ADOA. Autophagy is critically important for optic nerve survival (Rodríguez-Muela et al., 2012) and increased mitophagy may generate cellular demands that are important in neurodegeneration. This model will thus be useful for further studies of neurodegeneration caused by impaired mitochondrial dynamics.

## Author contributions

AD, TA, JS, CL, JC, RN, RG, YG, CW, SS, MD, ED, SW, TE and MV performed the experiments. JP, KM, and AD designed the experiments. JP, JS, TL, FI, KM, and AD analyzed the results. JP, AD, JS, KM, and MD wrote this manuscript.

### Conflict of interest statement

The authors declare that the research was conducted in the absence of any commercial or financial relationships that could be construed as a potential conflict of interest.

## Supplementary material

Figure S1 Mapping of the mRFP transgene. The insertion of the mRFP transgene was investigated by paired-end sequencing. The insert is located in the 3rd exon of the *pkn1* gene, which has been confirmed by PCR using a common forward primer (green) and two different reverse primers; one (red) 1,500 bp from the forward primer in the wild type genome (blue) and one in the mRFP insert, approximately 800 bp from the forward primer. The 1,500 bp PCR only works in the wild type genome and the 800 bp only when the insert is present.

Figure S2 Analysis of OPA1 expression in the MEFs used. Protein extracts were prepared from wt, RedMIT-GFP-LC3 and RedMIT-GFP-LC3-OPA1^Q285STOP^ MEFs and analyzed on a 8% acrylamide gel. The OPA1 signal was detected using a rabbit anti-OPA1 antibody (abcam ab42364) and revealed using a polyclonal goat anti-rabbit secondary antibody (Dako P0448) coupled to an ECL detection kit (WESTAR^®;^ Supernova HRP Detection Substrate, Geneflow K1-0068) according to the manufacturer's instructions and imaged with G:BOX (Syngene).

Figure S3 Images from sections of brain and spleen from control, RedMIT/GFP-LC3, and OPA1^Q285STOP^/RedMIT/GFP-LC3. Brain **(Bottom)** and spleen **(Top)** from non-fluorescent (control), RedMIT/GFP-LC3 and OPA1^Q285STOP^/RedMIT/GFP-LC3 mice were sectioned (10 μm) and imaged on a Zeiss LSM 700 inverted confocal microscope with a plan-Apo 63x NA 1.4 oil-immersion objective. The fluorescent signals, GFP-LC3 and mitochondrial mRFP, are visible but no clear colocalization between them could be visualized in the views shown. High throughput imaging identified infrequent colocalization that was more marked in OPA1^Q285STOP^/RedMIT/GFP-LC3 mice than RedMIT/GFP-LC3 mice (Figure 5). Scale bars = 10 μm.

Table S1 mRFP and GFP transgenes do not affect mice reproductive success.

Video S1 Movie of the RedMIT-GFP-LC3 MEFs. Live RedMIT-GFP-LC3 MEFs have been imaged using a custom Olympus IX81 inverted microscope equipped with temperature control (Solent scientific) every 30 s for 8 h. This can be used to quantify the early stages of mitophagy in real time.
