# Supplementary figures and images for: Validating the RedMIT/GFP-LC3 Mouse Model by Studying Mitophagy in Autosomal Dominant Optic Atrophy Due to the OPA1Q285STOP Mutation
Source: Front Cell Dev Biol. 2018 Sep 19;6:103. doi: 10.3389/fcell.2018.00103 (PMC6156146; doi:10.3389/fcell.2018.00103)

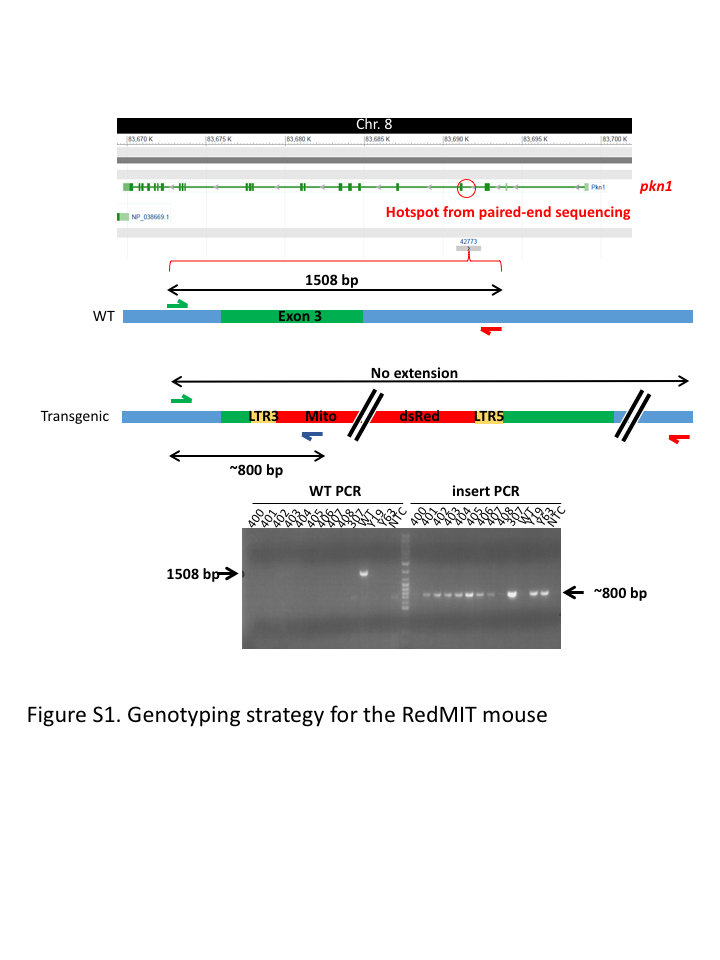

Supplement: Figure S1 — Mapping of the mRFP transgene. The insertion of the mRFP transgene was investigated by paired-end sequencing. The insert is located in the 3rd exon of the pkn1 gene, which has been confirmed by PCR using a common forward primer (green) and two different reverse primers; one (red) 1,500 bp from the forward primer in the wild type genome (blue) and one in the mRFP insert, approximately 800 bp from the forward primer. The 1,500 bp PCR only works in the wild type genome and the 800 bp only when the insert is present. [file Image_1.tiff]

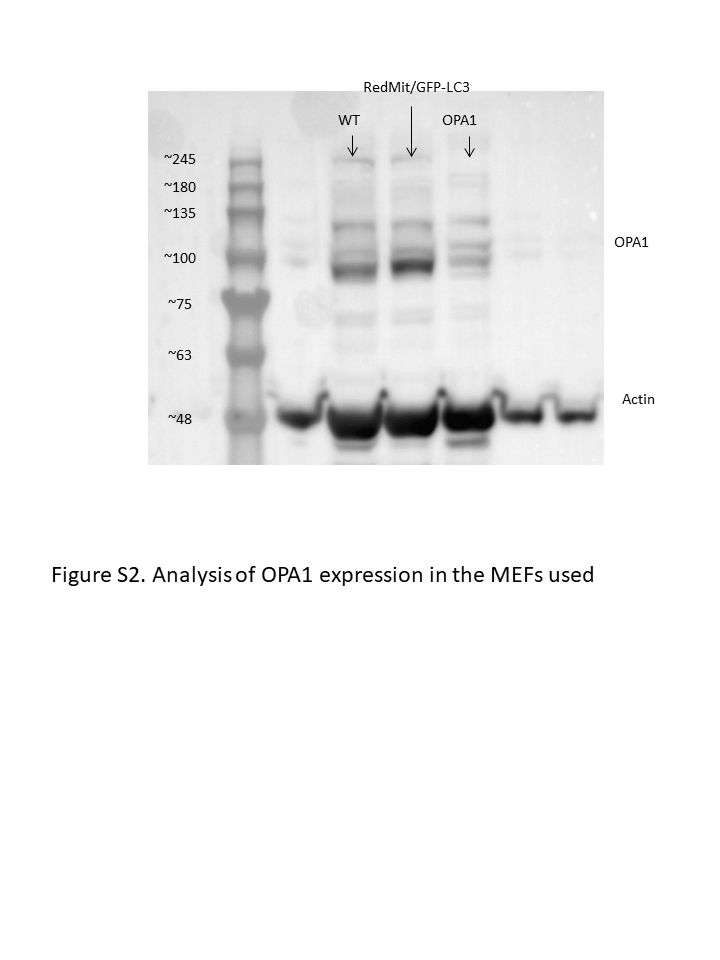

Supplement: Figure S2 — Analysis of OPA1 expression in the MEFs used. Protein extracts were prepared from wt, RedMIT-GFP-LC3 and RedMIT-GFP-LC3-OPA1Q285STOP MEFs and analyzed on a 8% acrylamide gel. The OPA1 signal was detected using a rabbit anti-OPA1 antibody (abcam ab42364) and revealed using a polyclonal goat anti-rabbit secondary antibody (Dako P0448) coupled to an ECL detection kit (WESTAR®; Supernova HRP Detection Substrate, Geneflow K1-0068) according to the manufacturer's instructions and imaged with G:BOX (Syngene). [file Image_2.tif]

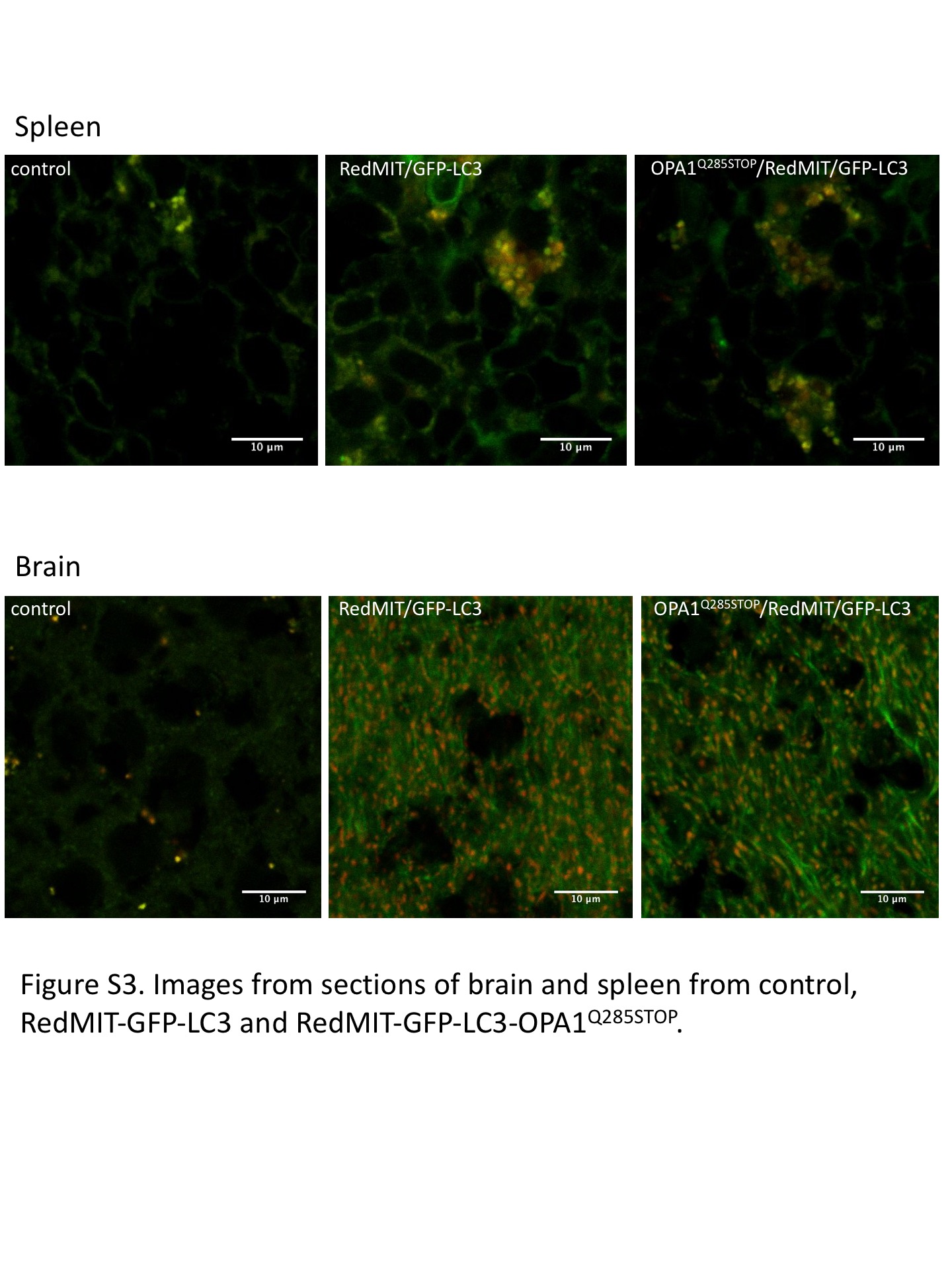

Supplement: Figure S3 — Images from sections of brain and spleen from control, RedMIT/GFP-LC3, and OPA1Q285STOP/RedMIT/GFP-LC3. Brain (Bottom) and spleen (Top) from non-fluorescent (control), RedMIT/GFP-LC3 and OPA1Q285STOP/RedMIT/GFP-LC3 mice were sectioned (10 μm) and imaged on a Zeiss LSM 700 inverted confocal microscope with a plan-Apo 63x NA 1.4 oil-immersion objective. The fluorescent signals, GFP-LC3 and mitochondrial mRFP, are visible but no clear colocalization between them could be visualized in the views shown. High throughput imaging identified infrequent colocalization that was more marked in OPA1Q285STOP/RedMIT/GFP-LC3 mice than RedMIT/GFP-LC3 mice (Figure 5). Scale bars = 10 μm. [file Image_3.jpg]
