# Supplementary material for: Validating the RedMIT/GFP-LC3 Mouse Model by Studying Mitophagy in Autosomal Dominant Optic Atrophy Due to the OPA1Q285STOP Mutation
Source: Front Cell Dev Biol. 2018 Sep 19;6:103. doi: 10.3389/fcell.2018.00103 (PMC6156146; doi:10.3389/fcell.2018.00103)
Supplement: Table S1 — mRFP and GFP transgenes do not affect mice reproductive success. [file Table_1.pdf]

Strain	Reproductive index	Pre-wean mortality
RedMIT/GFP-LC3	0.52	31%
RedMIT/GFP-LC3/OPA1	0.59	25%
OPA1+/-	0.56	25%

Table S1. mRFP and GFP transgenes do not affect mice reproductive success
